# The aging kidney is characterized by tubuloinflammaging, a phenotype associated with MHC-II gene expression

**DOI:** 10.3389/fimmu.2023.1222339

**Published:** 2023-08-22

**Authors:** Julius Sinning, Nils David Funk, Inga Soerensen-Zender, Vera Christine Wulfmeyer, Chieh Ming Liao, Hermann Haller, Christian Hinze, Kai Martin Schmidt-Ott, Anette Melk, Roland Schmitt

**Affiliations:** ^1^ Department of Nephrology and Hypertension, Hannover Medical School, Hannover, Germany; ^2^ Department of Pediatric Kidney, Liver and Metabolic Diseases, Hannover Medical School, Hannover, Germany

**Keywords:** aging kidney, senescence, tubular cell, epithelial cell, inflammation, MHC-II, CD74

## Abstract

**Introduction:**

Even during physiologic aging, the kidney experiences a loss of mass and a progressive functional decline. This is clinically relevant as it leads to an increased risk of acute and chronic kidney disease. The kidney tubular system plays an important role in the underlying aging process, but the involved cellular mechanisms remain largely elusive.

**Methods:**

Kidneys of 3-, 12- and 24-month-old male C57BL/6J mice were used for RNA sequencing, histological examination, immunostaining and RNA-in-situ-hybridization. Single cell RNA sequencing data of differentially aged murine and human kidneys was analyzed to identify age-dependent expression patterns in tubular epithelial cells. Senescent and non-senescent primary tubular epithelial cells from mouse kidney were used for in vitro experiments.

**Results:**

During normal kidney aging, tubular cells adopt an inflammatory phenotype, characterized by the expression of MHC class II related genes. In our analysis of bulk and single cell transcriptional data we found that subsets of tubular cells show an age-related expression of Cd74, H2-Eb1 and H2-Ab1 in mice and CD74, HLA-DQB1 and HLADRB1 in humans. Expression of MHC class II related genes was associated with a phenotype of tubular cell senescence, and the selective elimination of senescent cells reversed the phenotype. Exposure to the Cd74 ligand MIF promoted a prosenescent phenotype in tubular cell cultures.

**Discussion:**

Together, these data suggest that during normal renal aging tubular cells activate a program of ‘tubuloinflammaging’, which might contribute to age-related phenotypical changes and to increased disease susceptibility.

## 1 Introduction

For maintaining the inner milieu, the kidney uses a complex system of filtration, reabsorption and secretion, which needs to work reliably for a lifetime. Filtration happens in the glomeruli while the urinary processing takes place in the renal tubular system. Aging leads to the loss of glomeruli and to atrophy and functional alterations of tubular cells (1). Due to their highly energy-consuming task of reabsorption and secretion, renal tubular cells are particularly vulnerable to stress and are often the center of acute kidney damage. It is therefore not surprising that tubular damage and maladaptive tubular repair are important contributors to the progression of chronic kidney disease (CKD), a condition characterized by chronic loss of filtration, interstitial fibrosis, and tubular atrophy (2). Pushed by the enormous therapeutic success of SGLT2-inhibitors, which block the uptake of glucose and sodium in the proximal tubule, the identification of novel target mechanisms in tubular cells has become an important strategic goal for innovative therapies (3).

In situations of excessive stress, tubular cells activate a damage program, characterized by the expression of pro-inflammatory cytokines and chemokines. This program mediates active crosstalk with professional immune cells, thereby promoting renal inflammation and injury. Besides the secretion of cytokines and chemokines, tubular cells may adopt features of professional immune cells, including the expression of major histocompatibility complex class II (MHC-II) genes like Cd74 and H2-Ab1 (4–6). The significance of this phenomenon in tubular cells has recently been demonstrated as mice with genetic tubule-specific ablation of H2-Ab1 showed a strongly improved phenotype in pro-fibrotic damage models (5).

Among the known mechanisms of renal aging, cellular senescence plays a crucial role in the tubular epithelium (7, 8). Cellular senescence is a stress response, in which cells are permanently cell cycle arrested, while they remain metabolically active. Most senescent cells have a particular secretory profile, which may vary according to the affected cell-type and the context of senescence induction. The characteristic secretion of bioactive molecules, known as the senescence associated secretory phenotype (SASP), typically comprises the synthesis of diverse cytokines, chemokines, growth factors, and proteases (8). Secretion of these factors can have beneficial effects during early phases of life (e.g. organ patterning) and in acute wound repair but when chronically secreted the same factors may disrupt normal tissue function and promote organismal aging and age-related diseases. Senescent cells accumulate in the kidney in advanced age and with chronic disease and there is growing experimental evidence that antagonizing senescence can harness old or stressed kidneys against injury, fibrosis and CKD progression (9–11).

Here, we investigate features of kidney aging with a focus on tubular cells. We speculated that there might be an association between natural tubular aging, cellular senescence and the development of inflammatory features, so far only described in acute kidney disease (i.e. MHC-II gene expression). In order to test this, we analyzed bulk and single cell transcriptomic data of kidneys from healthy aged mice and humans and tested the impact of senescence induction and senescence elimination on inflammatory features in cultured tubular cells.

## 2 Materials and methods

### 2.1 Mice for RNAseq and histological analysis

3-, 12- and 24-month-old male C57BL/6JRj mice (Janvier, Le Genest-Saint-Isle, France) were used for bulk-RNA sequencing experiments. After euthanization, one half of the right kidney was snap frozen for RNA extraction, while the other half was immersion fixed in 4% paraformaldehyde for histological evaluation. Standard histological analysis was performed using hematoxylin eosin stained 4 µm sections. Only kidneys without macroscopic or histological pathology (e.g. ureteral obstruction, amyloidosis) were included. All animal experiments were done in agreement with institutional and legislator regulations and approved by the local authorities.

### 2.2 Bioinformatical analysis of bulkRNAseq and scRNAseq datasets

RNA extraction from primary tubular epithelial cells (PTEC) and murine kidneys for RT-qPCR and bulk sequencing was performed using NucleoSpin RNA Plus kits (Macherey-Nagel). 250 ng of RNA per sample was used for mRNA enrichment and cDNA library generation, following sequencing on an Illumina Next Seq 550 sequencer. Generated BCL files were converted into FASTQ files. Raw data processing was followed by data normalization using DESeq2. The detailed sequencing process is described in the **Supplemental Methods**. Normalized data was used for Gene Set Enrichment Analysis (GSEA) (12). Terms with a FDR/q-value < 0.05 were defined as significant. To identify Gene Ontology Terms enriched *in vitro* as well as *in vivo*, MSigDB GSEA analysis including genes that were significantly up- or down-regulated (FDR < 0.05, log2FC > 0.5) in both data sets (*in vivo and in vitro*) was performed (13). Processed bulk RNAseq data sets from the Tabula Muris Senis consortium (14) (age 3, 6, 9, 12, 24 months) were used in order to analyze MHC-II expression in kidneys from male mice during aging (https://twc-stanford.shinyapps.io/maca/). Processed droplet scRNAseq kidney data sets from the Tabula Muris Senis consortium was downloaded at https://figshare.com/articles/dataset/Tabula_Muris_Senis_Data_Objects. SCANPY (15) was used for further analysis and data visualization. For identification of senescent cells, proximal tubular cells were filtered (‘epithelial cell of proximal tubule’, ‘kidney proximal convoluted tubule epithelial cell’, ‘kidney proximal straight tubule epithelial cell’) and re-clustered using the Louvain package (neighbors = 10, PCA = 10, perplexity = 20, resolution = 1). Senescence enriched clusters were identified by the combination of increased Cdkn1a (p21) expression in combination with low abundance of cells in S phase and additional analysis of senescence-associated GSEA terms using GSEApy package and the Reactome pathway database. Unbiased DEG (differential expressed genes) analysis was performed using diffxpy package. Processed scRNAseq data sets from the KPMP consortium were downloaded at atlas.kpmp.org (16). h5Seurat-files were converted to.h5ad-files using Seurat (17). Cells from living donors and CKD patients were filtered and used for further analysis. Identification of senescent cells was performed as described above using SCANPY.

### 2.3 Cell culture experiments

PTEC were isolated as described previously (18, 19). In brief, kidneys from C57BL/6JRj mice (Janvier, Le Genest-Saint-Isle, France) were digested in 0.125% Collagenase Type I (Affymetrix/USB) solution at 37°C for 45 minutes. Tubular fragments were sedimented, sieved through 40 µm cell strainers and cultured in REGM2 medium (Promocell). Confluent PTEC were exposed to γ-irradiation (γ-ray, 10 Gray) to induce synchronized senescence. For senolytic treatment, PTEC were treated with 0.5µM/ml ABT-263 (MCE, NY, USA) for 24 hours before harvest. Irradiated cells were also treated with MIF (1978-MF-0257CF, R&D Systems, Minneapolis, MN) at a concentration of 200ng/ml. Medium was changed every 48 hours and cells were harvested after 10 days. Senescence-associated-ß-Galactosidase (SA-ß-Gal) assessment was performed as described previously (18).

### 2.4 In-situ-hybridization and immunofluorescence

RNA In-Situ-Hybridization was performed according to the RNAscope^®^ 2.5 HD Detection Kit (BROWN) Quick Guide (20). In brief, formalin-fixed paraffin-embedded kidneys were cut in 5µm sections and mounted on Superplus^®^ Frost slides. Samples were deparaffinized followed by target retrieval steps. Purchased probes for Cd74 (Cat No. 437501) and H2-Eb1 (Cat No. 509081) were used for hybridization. For additional immunostaining, sections were stained with primary anti-Lrp2 antibody (Abcam, Cambridge, UK). Antibody visualization was achieved using Alexa Fluor 488 anti-rabbit antibody (Molecular Probes/Invitrogen, Carlsbad, CA, USA). Pictures were taken with a Leica DM IRB with a TCS SP2 AOBS scan head (Leica, Jena, Germany).

### 2.5 Real-time RT-PCR

RNA from cells or kidney tissue was isolated using NucleoSpin RNA Plus kits (Machery-Nagel). After reverse transcription, mRNA expression was determined using a Lightcycler 480 System (Roche) with SYBR green master mix and specific primers (**Supplemental Table 1**) For quantitative analysis, relative mRNA levels were calculated according to the 2-ΔCt method; samples were normalized to Hprt gene expression.

### 2.6 Statistical analysis

Results are expressed as means ± SEM unless otherwise indicated. Statistical significance was calculated by unpaired two-tailed t-test for comparison of two groups, one-way or two-way ANOVA followed by Tukey’s *post hoc* test for multiple comparisons using GraphPad Prism^®^ Software. For t-test p < .05 was considered as statistical significance. FDR/q-values and log2FC generated by DESeq2 were used for bulkRNAseq analysis, FDR/q-values and log2FC generated by diffxpy were used for scRNAseq, considering FDR/q-values < 0.05 and log2FC > 0.5 as significant.

### 2.7 Data availability

The RNAseq datasets generated for this study can be found in the Gene Expression Omnibus (GEO) database (GEO Accession ID: GSE233718).

## 3 Results

### 3.1 Cellular senescence is associated with MHC-II gene expression in tubular cells *in vitro*

**Figure 1:**
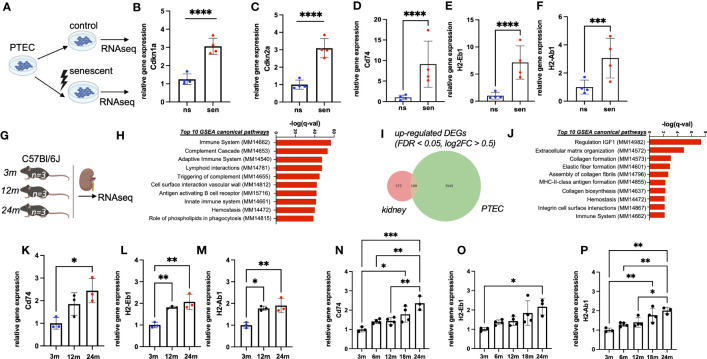
**(A)** Schematic of the generation of RNAseq data sets from senescent (sen) and non-senescent (ns) primary tubular epithelial cells (PTEC) (n=4). **(B, C)** Quantification of transcripts of senescence marker genes Cdkn1a (p21) and Cdkn2a (p16^INK4a^) by RNAseq data from PTEC. **(D–F)** Quantification of transcripts of MHC-II related genes Cd74, H2-Ab1 and H2-Eb1 by RNAseq data from PTEC. **(G)** Schematic of the generation of RNAseq data sets from kidneys of three, 12 and 24 months (m) old mice (each group n=3). **(H)** Bar diagram showing upregulation of immune-related pathways by GSEA. **(I)** Venn diagram showing upregulation of 109 overlapping differentially expressed genes (DEGs) in both data sets generated by RNAseq. **(J)** Bar diagram showing enriched pathways of overlapping genes by GSEA. **(K–M)** Quantification of transcripts of MHC-II related genes Cd74, H2-Ab1 and H2-Eb1 by RNAseq data from mice. **(N–P)** Quantification of transcripts for MHC-related genes Cd74, H2-Ab1 and H2-Eb1 by RNAseq data from Tabula muris senis. Results are presented as means ± SEM of at least three repeats for each experiment. Significance was tested by FDR/q-value generated by DESeq2, one-way ANOVA, or two-way ANOVA with Tukey’s test as *post hoc* analysis in the case of multiple comparisons. ns, non-senescent; *p < .05; **p < .01; ***p < .001, ****p < .0001.

In an *in vitro* aging model primary tubular epithelial cells (PTEC) of C57Bl/6J mice were treated by γ-irradiation to induce cellular senescence (**Figure 1A**). Cellular senescence was confirmed by the up-regulation of typical senescence markers Cdkn1a (p21) and Cdkn2a (p16^INK4a^) (**Figures 1B, C**) and quantification of SA-ß-Gal staining (**Supplemental Figures 1A-C**). Analysis of senescent versus non-senescent cells revealed a strong induction of immune pathway genes in senescent PTEC, including the up-regulation of MHC-II genes Cd74, H2-Eb1 and H2-Ab1 (**Figures 1D–F**). Of note, genes for typical tubular injury markers, such as Havcr1 (Kim-1) and Krt8, Krt18, Krt19, Krt7 (tubular keratins) were not up- but rather down-regulated (**Supplemental Figures 1D–H**).

### 3.2 Chronological kidney aging is associated with MHC-II gene expression

To study the expression of MHC-II genes *in vivo* we performed bulk RNA sequencing of kidneys from healthy male mice at three different ages (three months, 12 months, 24 months; **Figure 1G**). We observed a clear age-dependent up-regulation of immune related genes (**Figure 1H**). Among differentially expressed transcripts, we found 109 up-regulated genes shared between transcriptomes of kidney homogenates and isolated senescent PTEC (**Figure 1I**). MHC-II genes Cd74, H2-Eb1 and H2-Ab1 were among the most up-regulated transcripts and were represented in the top gene ontology terms (**Figures 1J–M**). A similar up-regulation was independently confirmed in the bulk RNA sequencing data set of the Tabula Muris Senis consortium (14). In this cohort, we found a clear increase in renal Cd74, H2-Ab1 and H2-Eb1 expression over the two-year lifespan of C57Bl/6 mice (**Figures 1N–P**). Importantly, in our bulk RNA sequencing we also observed a strong correlation with aging for increased expression of Cd74, H2-Ab1 and H2-Eb1, whereas canonical immune cell marker genes Adgre1, Cd81 and Ptprc were just slightly up-regulated (**Supplemental Figures 2A–F**).

### 3.3 Renal tubular cells express MHC-II genes during normal kidney aging

**Figure 2:**
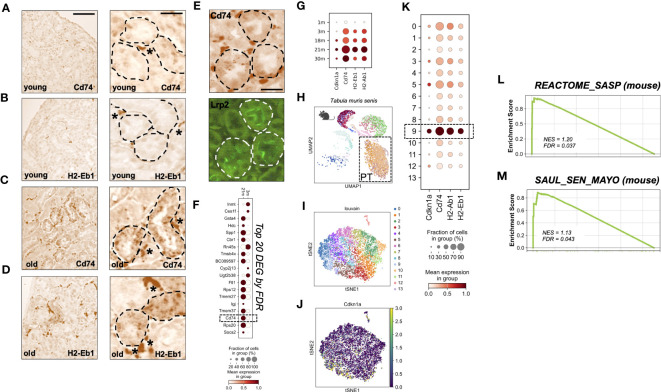
**(A–D)** RNA *in-situ* hybridization of MHC-II related genes Cd74 and H2-Eb1 in three month old = young **(A, B)** and 24 month old = old kidneys **(C, D)**, scale bar: 100µm; 30µm. * marks stained interstitial cells. **(E)** Immunofluoresence co-staining using Lrp2-antibody (Megalin), scale bar: 30µm. **(F)** Dot plot showing top 20 up-regulated genes comparing young (3m) and old (21m) proximal tubular cells ordered by FDR/q-value. **(G)** Dot plot showing age-dependent expression of MHC-II related genes Cd74, H2-Ab1, H2-Eb1 and senescence marker gene Cdkn1a (p21). **(H)** Uniform Manifold Approximation and Projection for Dimension Reduction (UMAP) showing scRNAseq kidney data set from Tabula muris senis. **(I)** tSNE plot showing reclustered proximal tubular cells (encircled in H) using louvain. **(J)** tSNE plot showing Cdkn1a expression. **(K)** Dot plot reclustering of proximal tubular cells identified 14 clusters. Senescent cluster 9 is labelled by rectangle. **(L, M)** Enrichment plots showing up-regulation of senescence-associated Gene Ontology terms by GSEA.

To investigate the local expression of MHC-II genes on a cellular level, we performed mRNA *In situ* hybridization for Cd74 and H2-Eb1 in kidneys of young and aged mice. In young kidneys, expression was exclusively found in interstitial cells, i.e. the typical location of mononuclear phagocytes/macrophages (**Figures 2A, B**). In contrast, old kidneys showed additional expression of Cd74 and H2-Eb1 in a subset of tubular cells (**Figures 2C, D**). Double labeling revealed that the tubular signals were mostly found in co-localization with the proximal tubular (PT) marker Megalin (Lrp2) (**Figure 2E**).

Next, we analyzed the expression of MHC-II genes in the Tabula Muris Senis single cell transcriptome data set (14). Unbiased DEG analysis of PT cells from three and 21 months old mice revealed that Cd74 was among the top 20 up-regulated DEGs ordered by FDR/q-value (**Figure 2F**). Up-regulation of MHC-II related genes was also reflected by a stepwise increase in different age-strata (**Figure 2G**). As described by others in single cell data sets, we found that Cdkn2a (p16^INK4a^) transcript levels were too low for reliable detection of cellular senescence (21, 22). To examine senescence on the single cell level we therefore used an algorithm described by O’Sullivan et al., which relies on Cdkn1a (p21) expression for enrichment of senescent cells in combination with low abundance of cells in S phase (22). One out of 14 PT sub-clusters met these senescence-criteria (**Figures 2H–K**). In this senescence-enriched PT cluster (cluster nine), expression of MHC-II genes Cd74, H2-Eb1 and H2-Ab1 was above average (**Figure 2K**). The state of senescence in this cluster was corroborated by a significant increase of the murine SASP Reactome pathway (**Figure 2L**) and the SenMayo signature, a novel high fidelity marker gene set for senescent cells (21) (**Figure 2M**).

### 3.4 Age-dependent MHC-II gene expression in tubular cells of aged human kidneys

**Figure 3:**
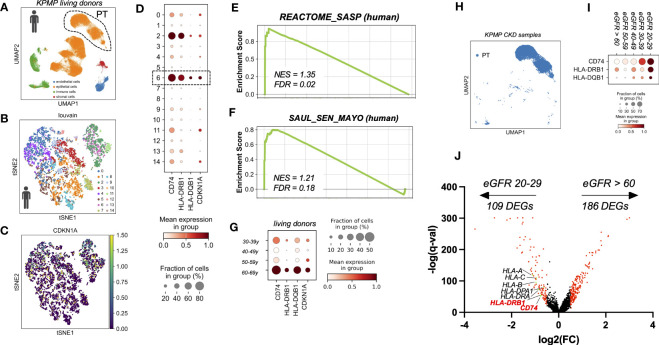
**(A)** UMAP showing scRNAseq living donor kidney data set from the Kidney Precision Medicine Project (KPMP project). **(B)** tSNE plot showing reclustered proximal tubular cells using louvain. **(C)** tSNE plot showing CDKN1A expression. **(D)** Dot plot reclustering of proximal tubular cells identified 15 clusters. Senescent cluster 6 is labelled by rectangle. **(E, F)** Enrichment plots showing up-regulation of senescence-associated Gene Ontology terms by GSEA. **(G)** Dot plot showing age-dependent expression of MHC-II related genes CD74, HLA-DQB1, HLA-DRB1 and senescence marker gene CDKN1A (p21). **(H)** UMAP showing proximal tubular cells (PT) of CKD samples (defined by eGFR < 60 ml/min) from the KPMP project. **(I)** Dot plot showing eGFR-dependent up-regulation of MHC-II related genes CD74, HLA-DRB1 and HLA-DQB1. **(J)** Volcano plot showing gene expression from unbiased DEG analysis using diffxpy.

To test whether the findings from murine kidneys also apply to human kidney aging, we used single cell RNA sequencing data of healthy kidneys from living transplant donors of KPMP consortium (16). With a focus on PT cells (**Figures 3A, B**) we observed, that similar to mice, a subset of human PT cells showed an age-dependent up-regulation of CDKN1A (p21) (**Figures 3C, D**), which was accompanied by increased expression of MHC-II related genes, including the human orthologous genes CD74, HLA-DQB1 and HLA-DRB1 (**Figure 3G**). Identification of senescence-enriched PT cell clusters based on CDKN1A (p21) expression and low abundance of S phase, revealed one relevant sub-cluster (cluster six) (**Figure 3D**). Senescence enrichment within these sub-clusters was supported by increased expression of human SenMayo gene set and significant up-regulation of human SASP Reactome. (**Figures 3E, F**). Importantly, compared to all other PT clusters the senescence-enriched sub-cluster showed the highest expression levels of CD74, HLA-DQB1 and HLA-DRB1 (**Figure 3D**). These data further indicate a link between the expression of MHC-II related genes and the state of cellular senescence in human PT cells *in vivo*. Furthermore, unbiased DEG analysis comparing PT cells from CKD samples by estimated GFR (eGFR 20-29 ml/min versus eGFR > 60 ml/min) revealed a significant up-regulation of multiple HLA-related genes including CD74 and HLA-DRB1 in kidneys with reduced filtration (**Figures 3H–J**).

### 3.5 Senolysis reduces MHCII gene expression while exposure to the Cd74 ligand MIF promotes a pro-senescent state

**Figure 4:**
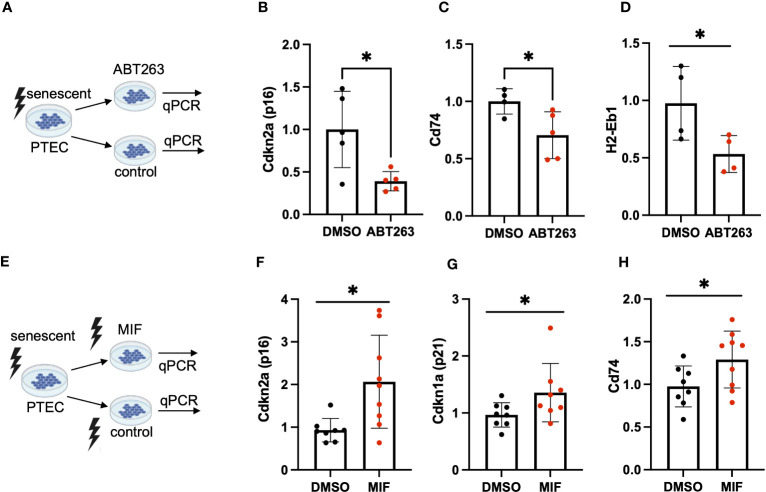
**(A)** Schematic of ABT-263 treatment in PTEC. **(B–D)** Quantification of transcripts for senescence marker Cdkn2a (p16^INK4a^), MHC-II related genes Cd74, H2-Eb1 in ABT-263-treated PTEC and controls by qPCR. **(E)** Schematic of MIF treatment in PTEC. **(F–H)** Quantification of transcripts for senescence markers Cdkn1a (p21), Cdkn2a (p16^INK4a^) and MHC-II related Cd74 in MIF-treated PTEC and controls by qPCR. Results are presented as means ± SEM of at least three repeats for each experiment. Significance was tested by t-test; *p < .05.

To further elucidate a link between tubular cell MHC-II gene expression and cellular senescence, we employed senolysis, a strategy in which senescent cells are selectively killed, and tested for MHC-II gene expression in the surviving (non-senescent) cell population. To this end, we treated murine PTEC with the senolytic drug ABT263 (**Figure 4A**) and confirmed successful senolysis by a significant reduction of Cdkn2a (p16^INK4a^) (**Figure 4B**) and SA-ß-gal positivity (**Supplemental Figures 3A-C**). In parallel, we saw a reduction of Cd74 and H2-Eb1 expression (**Figures 4C, D**), suggesting that senolysis eliminated cells with higher expression of MHC-II genes. Cd74 is a high-affinity receptor for macrophage migration inhibitory factor (MIF), an inflammatory cytokine, involved in multiple signaling pathways. As MIF has been reported to prevent cellular senescence in mesenchymal stem cells (MSC) (23), we were interested in studying the effects of MIF on tubular cell senescence (**Figure 4E**). PTEC were exposed to MIF or vehicle and subsequently γ-irradiated to induce cellular senescence. We found that MIF exposure did not prevent senescence induction, but instead MIF had a pro-senescent effect as shown by enhanced expression of Cdkn2a (p16^INK4a^) and Cdkn1a (p21) (**Figures 4F, G**). The pro-senescent impact of MIF was accompanied by an up-regulation of Cd74 (**Figure 4H**), highlighting the interrelationship between cellular senescence, Cd74 and an inflammatory phenotype in tubular cells.

## 4 Discussion

In 2000 Franceschi et al. introduced the concept of inflammaging, which describes a chronic low-grade inflammation in older organisms (24). Here, we observed locally constricted inflammaging of the tubular system as an intrinsic cell response of aging. This phenomenon of ‘tubuloinflammaging’ was characterized by changes in the transcriptome including the expression of MHC-II genes in tubular cells of normally aged murine and human kidneys. While a similar pattern of tubular changes has previously been reported in the context of acute inflammatory kidney disease, our study is the first to link tubular low-grade inflammation to the natural aging process. With a focus on tubular cells our findings add to published aging studies, which described age-associated inflammation of the kidney with an influx of macrophages and a phenotypic switch of resident mesenchymal stromal cells (25, 26). Using isolated PTEC and single cell transcriptomic data, our approach allowed us to dissect the contribution of tubular cells and to specify the previously reported pro-inflammatory renal signature of aging (25, 27–30).

Our data indicate a functional intersection of cellular senescence and inflammation in the aging tubular system. Analysis of single cell transcriptome data revealed a strong association between markers of cellular senescence and the expression of MCH-II genes. After senolytic elimination of senescent tubular cells *in vitro*, we found that the surviving cells had significantly lower MHC-II levels. Similar to these findings, a study in melanocytes reported that oncogene induced senescence was accompanied by up-regulation of MHC-II gene expression and activation of the adaptive immune system (31). Along these lines, Breda et al. have shown in mice with nephrotoxic nephritis that tubular MHC-II expression resulted in proliferation and inflammatory cytokine production of immune cells (4). An analogous mechanism might be active in the aging kidney, where tubular MHC-II genes contribute to the age-related infiltration and activation of leukocytes.

MIF signaling can have heterogeneous effects, being either harmful or protective in the kidney depending on the clinical situation, the amount and time of MIF exposure and the stage of renal pathology (32–34). Interestingly, we observed that recombinant MIF promoted a pro-senescent phenotype in PTEC cultures, while Djudjaj et al. found that MIF knockout was associated with an increased stress-induced G2/M cell-cycle arrest of tubular cells (35). A recent single cell RNA sequencing study by Saul el al. identified MIF as a key SASP gene reflecting the burden of cellular senescence in hematopoietic and mesenchymal cells (21). Importantly, we could not find significantly elevated MIF transcription in aged kidneys or senescent tubular cells. However, as circulating MIF increases with aging (36), systemic exposure to age-related MIF levels may contribute to the development of tubular cell senescence. A recent genotyping study indicated that MIF polymorphisms, which cause a higher production of the protein, are significantly more frequent in patients with CKD (32). Given the growing interest in the development of MIF inhibiting therapies (33), our findings warrant further research as a pro-senescent role of MIF could offer a targetable mechanism in kidney aging and CKD.

From a technical point of view, our findings indicate a potential pitfall for cell type prediction by deconvolution algorithms. Computational deconvolution strategies have been developed to infer cell type proportions from bulk transcriptomic data (37). As the method relies on cell type specific marker gene expression, incorrect annotations result, if a cell type expresses atypical genes promiscuously. Along these lines, it is of note, that the top-scored genes for macrophage annotation include MHC-II genes Cd74, H2-Aa, H2-Ab1, H2-Eb1 (38, 39). Therefore, the expression of MHC-II genes by tubular cells may challenge the accuracy of predicting tubular cell and macrophage numbers when using a bulk RNA based deconvolution strategy.

A limitation of our study is the lack of functional experiments to elucidate the relationship between the expression of MHC-II related genes and kidney aging. Zhou et al. recently demonstrated by conditional knockout that stress induced H2-Ab1 expression in proximal tubular cells of younger mice aggravates kidney scarring while regulating important aspects of the renal damage response (5). Similar studies in aging mice will provide clues as to how cellular senescence and MHC-II related gene expression interconnect in tubular cells and how these processes contribute to the functional changes of renal aging.

In summary, our study revealed that the age-related phenotype of kidney tubular cells comprises the expression of Cd74, H2-Eb1 and H2-Ab1 in mice and CD74, HLA-DQB1 and HLA-DRB1 in humans. Expression of MHC-II related genes was associated with tubular cell senescence, which was reversed by selective elimination of senescent cells, whereas exposure to MIF promoted a pro-senescent phenotype. Although interventional experiments, which address the functional consequence of our observations, are still lacking, we propose that tubuloinflammaging might contribute to the decline and vulnerability of the aging kidney and might therefore be a potential therapeutic target.

## Data availability statement

The datasets presented in this study can be found in online repositories. The names of the repository/repositories and accession number(s) can be found below: GSE233718 (GEO).

## Ethics statement

The animal study was reviewed and approved by Niedersächsisches Landesamt für Verbraucherschutz und Lebensmittelsicherheit.

## Author contributions

Conceptualization: JS, AM, CH, RS. Methodology: JS, NF, IS-Z, CL, VW, CH, RS. Data analysis and interpretation: JS, IS-Z, CH, RS. Figure preparation: JS, VW, RS. Writing - original draft preparation: JS, RS. Writing - review and editing: NF, IS-Z, VW, CL, CH, KS-O, AM. All authors have read and approved the final version of the manuscript.

## Supplementary material

Supplementary Figure 1
**(A)** Quantification of Senescence-associated-ß-Galactosidase (SA-ß-Gal) positive area in PTEC comparing non-senescent and senescent cells. **(B, C)** Representative images showing SA-ß-Gal expression. **(D–H)** Quantification of transcripts of tubular injury marker genes Havcr1, Krt8, Krt18, Krt19, Krt7 by RNAseq data from non-senescent (ns) and senescent (sen) PTEC. FDR/q-value generated by DESeq2, *FDR < .05; **FDR < .01; ***FDR < .001, ****FDR<.0001.

Supplementary Figure 2
**(A–C)** Quantification of MHC-II related marker genes Cd74, H2-Eb1, H2-Ab1 in RNAseq data from mice. **(F–J)** Quantification of transcripts of immune cell marker genes Ptprc, Adgre1, Cd81 by RNAseq data from mice. FDR/q-value generated by DESeq2, *FDR < .05; **FDR < .01; ***FDR < .001, ****FDR<.0001.

Supplementary Figure 3
**(A)** Quantification of SA-ß-Gal positive area in PTEC comparing senescent and ABT263-treated senescent cells. **(B, C)** Representative images showing SA-ß-gal expression. Significance was tested by t-test. *p < .05; **p < .01; ***p < .001.
