# Supplementary figures and images for: The aging kidney is characterized by tubuloinflammaging, a phenotype associated with MHC-II gene expression
Source: Front Immunol. 2023 Aug 22;14:1222339. doi: 10.3389/fimmu.2023.1222339 (PMC10477980; doi:10.3389/fimmu.2023.1222339)

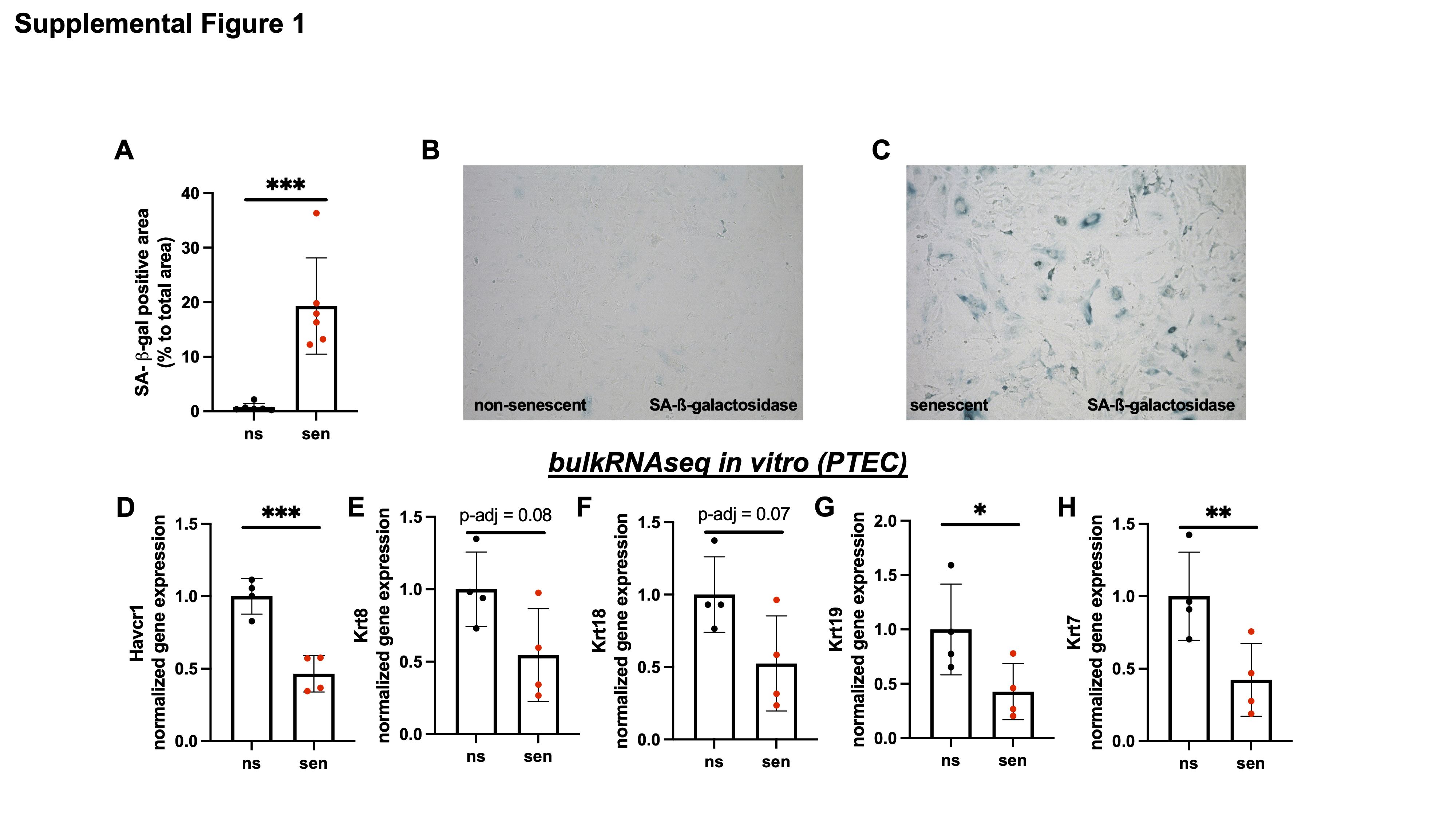

Supplement: Supplementary Figure 1 — (A) Quantification of Senescence-associated-ß-Galactosidase (SA-ß-Gal) positive area in PTEC comparing non-senescent and senescent cells. (B, C) Representative images showing SA-ß-Gal expression. (D–H) Quantification of transcripts of tubular injury marker genes Havcr1, Krt8, Krt18, Krt19, Krt7 by RNAseq data from non-senescent (ns) and senescent (sen) PTEC. FDR/q-value generated by DESeq2, *FDR < .05; **FDR < .01; ***FDR < .001, ****FDR<.0001. [file Image_1.jpeg]

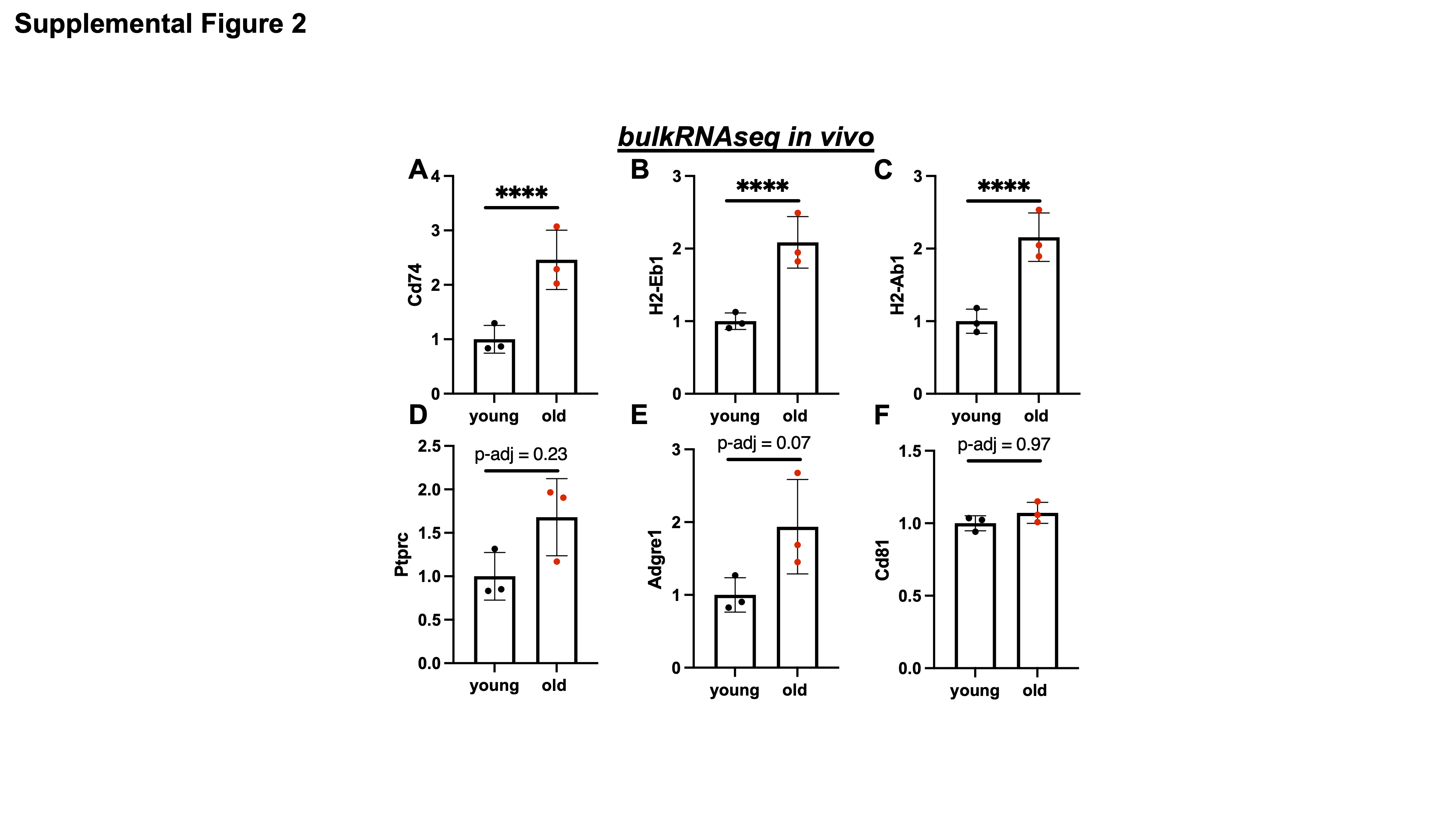

Supplement: Supplementary Figure 2 — (A–C) Quantification of MHC-II related marker genes Cd74, H2-Eb1, H2-Ab1 in RNAseq data from mice. (F–J) Quantification of transcripts of immune cell marker genes Ptprc, Adgre1, Cd81 by RNAseq data from mice. FDR/q-value generated by DESeq2, *FDR < .05; **FDR < .01; ***FDR < .001, ****FDR<.0001. [file Image_2.jpeg]

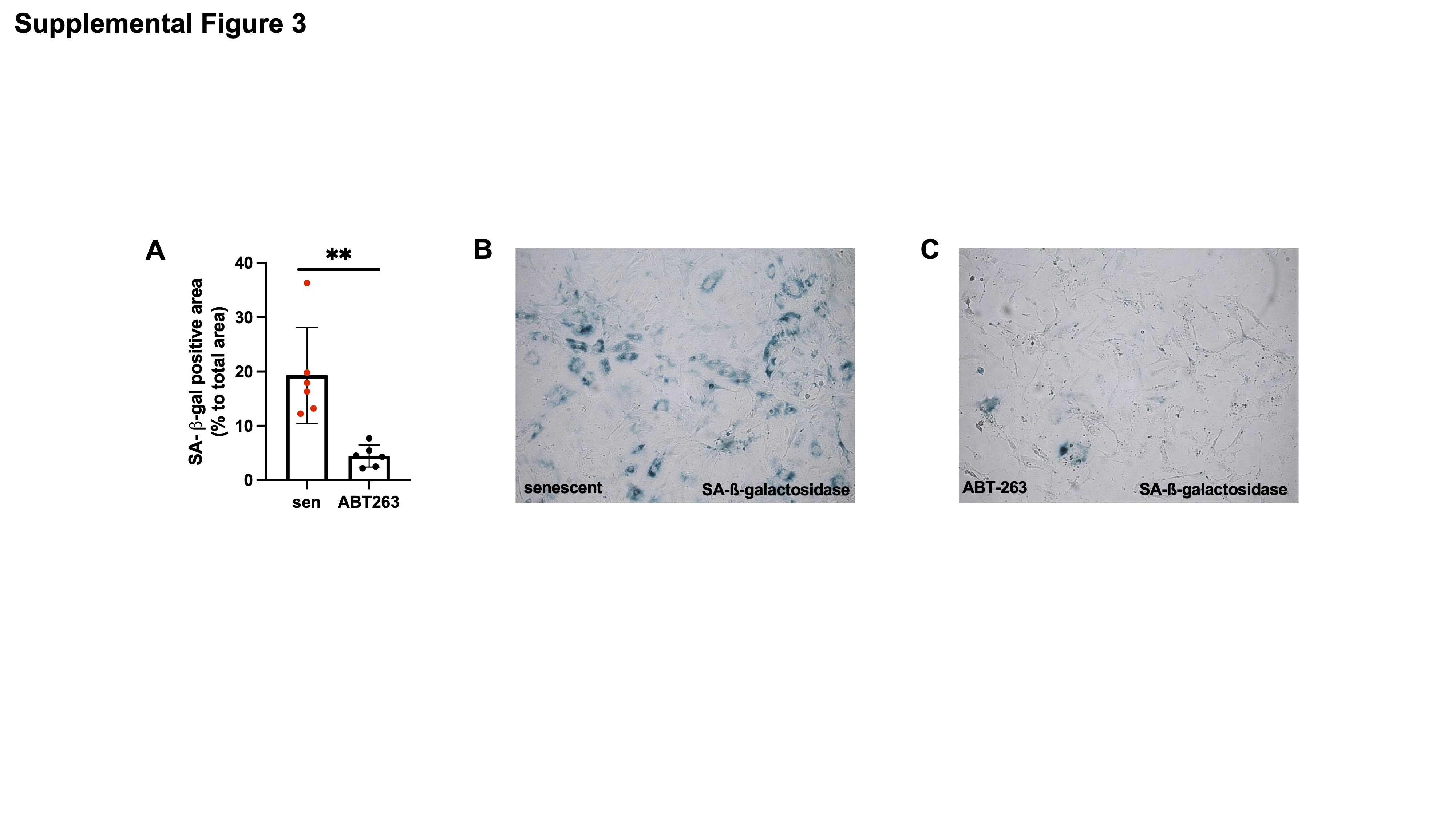

Supplement: Supplementary Figure 3 — (A) Quantification of SA-ß-Gal positive area in PTEC comparing senescent and ABT263-treated senescent cells. (B, C) Representative images showing SA-ß-gal expression. Significance was tested by t-test. *p < .05; **p < .01; ***p < .001. [file Image_3.jpeg]
