# Supplementary material for: The aging kidney is characterized by tubuloinflammaging, a phenotype associated with MHC-II gene expression
Source: Front Immunol. 2023 Aug 22;14:1222339. doi: 10.3389/fimmu.2023.1222339 (PMC10477980; doi:10.3389/fimmu.2023.1222339)
Supplement: Supplementary file 4 [file Table_1.pdf]

	forward primer
Cd74	5'-TGA CCA TCA CCT CCC AGA AC-3'
Cdkn1a	5'-CCA ATC TGC GCT TGG AGT GA-3'
Cdkn2a	5'-CGA ACT CTT TCG GTC GTA CCC-3'
Hprt	5'-TGA CAC TGG TAA AAC AAT GCA AAC T-3'
H2-Eb1	5'-GCG GAG AGT TGA GCC TAC G-3'

reverse primer
5'-GTA ACG TTC TTC ACA GGC CC-3'
5'-CTG TCT TGC ACT CTG GTG TCT GA-3'
5'-CGA ATC TGC ACC GTA GTT GAG C-3'
5'-AAC AAA GTC TGG CCT GTA TCC AA-3'
5'-AGG CCC GTG GAC ACA ATT C-3'
