# Supplementary material for: The aging kidney is characterized by tubuloinflammaging, a phenotype associated with MHC-II gene expression
Source: Front Immunol. 2023 Aug 22;14:1222339. doi: 10.3389/fimmu.2023.1222339 (PMC10477980; doi:10.3389/fimmu.2023.1222339)
Supplement: Supplementary file 5 [file DataSheet_1.docx]

**Library generation, sequencing, and raw data processing**

*Library generation, quality control, and quantification:*

250ng of total RNA per sample were utilized as input for mRNA enrichment procedure with ‘NEBNext® Poly(A) mRNA Magnetic Isolation Module’ (E7490L; New England Biolabs) followed by stranded cDNA library generation using ‘NEBNext® Ultra II Directional RNA Library Prep Kit for Illumina’ (E7760L; New England Biolabs). All steps were performed as recommended in user manualE7760 (Version 1.0_02-2017; NEB) except that all reactions were downscaled to 2/3 of initial volumes. Furthermore, one additional purification step was introduced at the end of the standard procedure, using 1x ‘Agencourt® AMPure® XP Beads’ (#A63881; Beckman Coulter, Inc.).

cDNA libraries were barcoded by dual indexing approach, using ‘NEBNext Multiplex Oligos for Illumina – 96 Unique Dual Index Primer Pairs’ (6440S; New England Biolabs). All generated cDNA libraries were amplified with 8 cycles of final pcr.

Fragment length distribution of individual libraries was monitored using ‘Bioanalyzer High Sensitivity DNA Assay’ (5067-4626; Agilent Technologies). Quantification of libraries was performed by use of the ‘Qubit® dsDNA HS Assay Kit’ (Q32854; ThermoFisher Scientific).

*Library denaturation and Sequencing run:*

Equal molar amounts of nine individually barcoded libraries were pooled. Accordingly, each analyzed library constitutes 11.1% of overall flowcell / run capacity. The library pool was denatured with NaOH and was finally diluted to 1.8pM according to the Denature and Dilute Libraries Guide (Document # 15048776 v02; Illumina). 1.3 ml of denatured pool were loaded on an Illumina NextSeq 550 sequencer. Two subsequent runs were performed and respective data were combined. Accordingly, two High Output Flowcells for 2x76bp paired-end reads (20024907; Illumina) were used in total. Sequencing was performed with the following settings: Sequence read 1 with 76 bases; sequence read 1 with 76 bases; Index reads 1 and 2 with 8 bases each.

*BCL to FASTQ conversion:*

BCL files were converted to FASTQ files using bcl2fastq Conversion Software version v2.20.0.422 (Illumina).

*Raw data processing and quality control:*

Raw data processing was conducted by use of nfcore/rnaseq (version 1.4.2) which is a bioinformatics best-practice analysis pipeline used for RNA sequencing data at the National Genomics Infrastructure at SciLifeLab Stockholm, Sweden. The pipeline uses Nextflow, a bioinformatics workflow tool. It pre-processes raw data from FastQ inputs, aligns the reads and performs extensive quality-control on the results. The genome reference and annotation data were taken from GENCODE.org (Mus musculus; GRCm38; release M18).

*Normalization and differential expression analysis (without outlier filtering):*

Normalization and differential expression analysis was performed with DESeq2 (Galaxy Tool Version 2.11.40.2) with default settings except for “Output normalized counts table”, “Turn off outliers replacement”, “Turn off outliers filtering”, and “Turn off independent filtering”, all of which were set to “True”.
